# Homocysteinylation of Fibrinogen: A Post-Translational Link to Thrombosis

**DOI:** 10.3390/ijms26125471

**Published:** 2025-06-07

**Authors:** Elvira Giurranna, Francesca Nencini, Serena Borghi, Ilenia Barbaro, Niccolò Taddei, Claudia Fiorillo, Matteo Becatti

**Affiliations:** Department of Experimental and Clinical Biomedical Sciences “Mario Serio”, University of Firenze, 50134 Firenze, Italy; elvira.giurranna@unifi.it (E.G.); francesca.nencini@unifi.it (F.N.); serena.borghi@unifi.it (S.B.); ilenia.barbaro@unifi.it (I.B.); niccolo.taddei@unifi.it (N.T.); claudia.fiorillo@unifi.it (C.F.)

**Keywords:** homocysteine, homocysteinylation, fibrinogen, thrombosis, oxidation, oxidative stress

## Abstract

Homocysteinylation, a post-translational modification involving the covalent attachment of homocysteine to proteins, has emerged as a critical mechanism linking hyperhomocysteinemia to thrombotic disease. This review focuses on the homocysteinylation of fibrinogen, a key coagulation factor, and its impact on clot structure and function. Evidence indicates that elevated homocysteine levels can induce significant changes in fibrin architecture, promoting the formation of dense, rigid clots with reduced permeability and impaired fibrinolytic susceptibility, thus fostering a prothrombotic environment. However, inconsistencies in reported effects on fiber diameter and polymerization kinetics highlight the need for standardized experimental protocols. Advances in proteomics and high-resolution imaging are expected to clarify the molecular underpinnings of these modifications. Moreover, homocysteinylation intersects with oxidative stress and may serve as a mechanistic bridge between metabolic and vascular dysfunction. Understanding its role not only enhances insight into thrombosis but also opens avenues for biomarker discovery and targeted therapies in cardiovascular and potentially neurological disorders.

## 1. Introduction

Homocysteine (Hcy) is a non-proteinogenic amino acid containing a sulfhydryl group, generated as an intermediate in the metabolic pathway that converts methionine (Met) to cysteine (Cys), rather than being directly obtained from the diet [1]. Under physiological conditions, Hcy does not accumulate in the body as it is either remethylated to Met or transsulfurated to Cys [2]. However, under pathological conditions, disruptions in this balance—caused by deficiencies in specific vitamins, enzymatic dysfunctions, or other factors—can lead to Hcy accumulation.

This review explores the biochemical and pathological roles of Hcy, with a particular emphasis on homocysteinylation, a prevalent post-translational modification of proteins. We examine how this modification alters protein structure and function, highlighting its implications in the pathogenesis of atherosclerosis, thrombosis, and inflammation. Special attention is given to fibrinogen, a key protein in blood coagulation, focusing on how its homocysteinylation influences clot formation, stability, and resistance to degradation, thereby increasing thrombotic risk.

A comprehensive literature review was conducted using the PubMed, Scopus, and Web of Science databases. Relevant publications were systematically screened to identify studies aligning with the objectives of this manuscript.

## 2. Homocysteine Metabolism

Hcy is generated in the body through transmethylation reactions during cellular metabolism, originating from Met, an essential amino acid obtained from dietary sources such as poultry, meat, eggs, seafood, and dairy products. Notably, Met serves as the sole dietary precursor of Hcy.

The liver plays a crucial role in Met and Hcy metabolism by providing the necessary enzymes to regulate plasma Hcy levels. Initially, Met absorbed from the diet is converted by Met adenosyltransferase into S-adenosylmethionine (SAM), a high-energy sulfonium compound. SAM then undergoes demethylation via methyltransferase enzymes, yielding S-adenosylhomocysteine (SAH), which is subsequently hydrolyzed by SAH hydrolase to produce Hcy and adenosine as a byproduct. Maintaining the metabolic balance of Hcy and its byproducts is essential for homeostasis in the human body [3,4].

**Figure 1:**
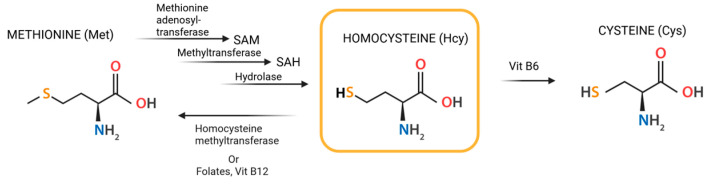
Structural formula of Hcy and its transformation.

Under normal physiological conditions, Hcy is either remethylated back to Met via the remethylation pathway—dependent on specific enzymes and vitamins, particularly folate (vitamin B9) and vitamin B12—or degraded into Cys through the trans-sulfuration pathway, which relies on vitamin B6 (Figure 1). Consequently, serum total Hcy levels are significantly influenced by the availability of these vitamins. Deficiencies in these vitamins or the enzymes involved in Hcy metabolism, as well as certain pathological conditions, can disrupt this balance [5,6].

Under normal physiological conditions, plasma Hcy levels range from 5 to 15 μmol/L. However, deficiencies in vitamin B6, vitamin B12, or folic acid, as well as genetic predispositions, can lead to elevated Hcy levels, a condition known as hyperhomocysteinemia (HHcy) [7]. HHcy is categorized based on severity: mild (15–30 μmol/L), moderate (30–100 μmol/L), and severe, where levels exceed 100 μmol/L [8].

HHcy and homocystinuria have been linked to various diseases, including cardiovascular disorders, inflammation, neurodegenerative diseases, chronic conditions, osteoporosis, depression, and pregnancy complications [8,9,10,11,12,13]. Several clinical studies have suggested an association between elevated Hcy levels and multiple pathological conditions [14,15,16,17,18]. Moreover, numerous studies have established that HHcy serves as a prognostic marker for various diseases [19,20,21,22,23].

The causes of HHcy include genetic mutations and enzyme deficiencies in 5,10-methylenetetrahydrofolate reductase (MTHFR), Met synthase (MS), and cystathionine β-synthase (CβS). Deficiencies in folate, vitamin B12, and, to a lesser extent, vitamin B6 can also disrupt Met metabolism, contributing to elevated Hcy levels [5]. Another key factor in HHcy is MTHFR gene mutation, which under normal conditions plays a crucial role in maintaining Hcy levels within the physiological range. Individuals with MTHFR mutations have an increased susceptibility to atherosclerosis and its associated complications, including myocardial infarction (MI), stroke, thrombotic events, and coronary artery disease [24,25]. Furthermore, HHcy can also result from a protein-rich diet and impaired renal function, both of which compromise the body’s ability to regulate and eliminate Hcy efficiently [5].

HHcy has detrimental effects on blood vessels, promoting the development of atherosclerosis and increasing the risk of thrombotic complications [24,26].

The molecular mechanisms by which Hcy contributes to the pathogenesis of cardiovascular diseases (CVDs) are complex and multifactorial [24,25].

The following are key processes through which Hcy exerts its pathological effects:

- Generation of Reactive Oxygen Species (ROS):

The proposed mechanisms underlying Hcy-induced oxidative stress [27,28,29,30,31,32] include (i) direct ROS formation via auto-oxidation in the presence of transition metals, (ii) the activation of pro-oxidant systems, and (iii) the inhibition of antioxidant defense mechanisms [33,34,35]. ROS, such as superoxide anions and hydrogen peroxide, are highly reactive molecules that damage endothelial cells, impairing their ability to maintain vascular integrity [36,37]. Additionally, Hcy can modify the structure and function of proteins by binding to their lysine (Lys) or Cys residues through post-translational modifications (PTMs) known as N-homocysteinylation and S-homocysteinylation, respectively. These mechanisms of Hcy-mediated injury are not mutually exclusive; alterations in protein expression and oxidative PTMs of proteins involved in pro-oxidant/antioxidant pathways can exacerbate oxidative stress, while, conversely, free radicals can induce changes in gene expression and oxidative PTMs [4]. Furthermore, ROS oxidize low-density lipoproteins (LDLs), promoting their accumulation in arterial walls—a key event in the development of atherosclerosis [38].

- Inhibition of Nitric Oxide (NO) Synthesis:

Hcy inhibits the activity of endothelial NO synthase (eNOS), the enzyme responsible for producing NO. NO is essential for vasodilation and endothelial protection. Reduced NO levels result in vasoconstriction, increased blood pressure, inflammation, and vascular damage, all of which contribute to the development of atherosclerosis [39].

- Activation of Cellular Receptors:

Hcy activates Toll-like receptor 4 (TLR4) and N-methyl-D-aspartate (NMDA) receptors. TLR4 activation triggers an inflammatory response by increasing the expression of pro-inflammatory cytokines such as interleukin-6 (IL-6) and tumor necrosis factor-alpha (TNF-α) [40,41,42]. Although NMDA receptors are primarily found in the nervous system, Hcy also activates them in vascular cells, leading to increased ROS production and endothelial dysfunction [43,44].

- Increased Inflammation:

Elevated Hcy levels are associated with the increased expression of pro-inflammatory cytokines, including interleukin-1β (IL-1β), IL-6, TNF-α, monocyte chemoattractant protein-1 (MCP-1), and intracellular adhesion molecule-1 (ICAM-1) [45,46,47]. IL-1β, IL-6, and TNF-α promote both systemic and localized inflammation. MCP-1 plays a crucial role in attracting monocytes to vascular walls, contributing to the formation of atherosclerotic plaques. Additionally, ICAM-1 facilitates leukocyte adhesion to endothelial cells, further exacerbating vascular damage and promoting endothelial dysfunction.

- Effects on gene expression:

Hcy alters the gene expression of proteins involved in cell adhesion and inflammation, thereby amplifying vascular damage and promoting the progression of atherosclerotic plaque formation [48,49,50]. Additionally, Hcy impairs epigenetic regulatory mechanisms of gene expression, including DNA methylation, histone modification, and non-coding RNA activity, which may further contribute to its toxicity [51]. These mechanisms collectively exacerbate vascular injury and significantly increase the risk of cardiovascular complications such as atherosclerosis, thrombosis, and hypertension. Given the complexity of these interactions, Hcy has emerged as a critical target for potential therapeutic interventions [13].

## 3. Homocysteinylation: Homocysteine Binding to Proteins

**Figure 2:**
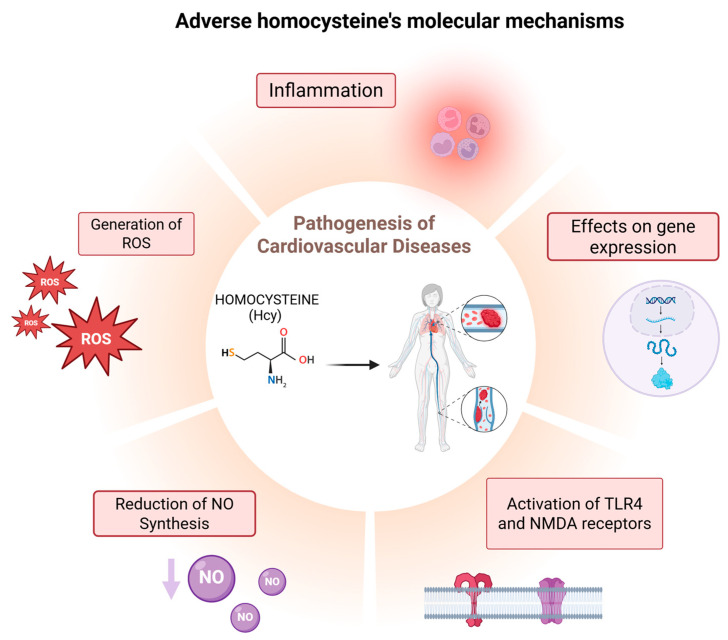
Diagram of the main molecular mechanisms through which Hcy contributes to the pathogenesis of CVD.

Epidemiological studies have established a strong association between elevated Hcy levels and adverse health outcomes, particularly cardiovascular and neurological diseases. First identified in the 1960s, severe HHcy has been implicated in significant neurological and cardiovascular impairments, often leading to premature mortality due to vascular complications [52] (Figure 2). While Hcy is a non-protein amino acid, recent studies have demonstrated its ability to incorporate into proteins, resulting in loss of function and the acquisition of cytotoxic, proinflammatory, proatherothrombotic, and proneuropathic properties, thereby contributing to various disease phenotypes linked to HHcy [53,54,55,56,57,58,59]. Under conditions of HHcy, Hcy undergoes abnormal binding to proteins through a process known as homocysteinylation. This post-translational modification (PTM) occurs after protein synthesis and significantly alters protein structure and function, further exacerbating pathological outcomes [60].

### Mechanisms of Protein Homocysteinylation

**Figure 3:**
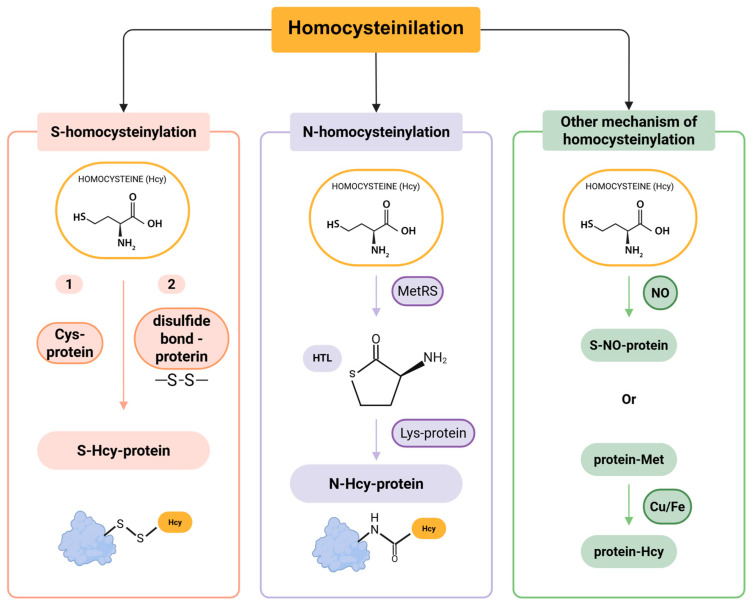
Pathways of protein homocysteinylation.

Homocysteinylation is a non-enzymatic chemical modification that occurs when Hcy or its toxic intermediate, homocysteine-thiolactone (HTL), interacts with proteins, leading to two distinct modifications: S-homocysteinylation and N-homocysteinylation [60]. These terms refer to the specific type of chemical bond formed between Hcy and a protein, depending on the involved atom. S-homocysteinylation occurs through the formation of a disulfide bond between the thiol group of Hcy and a Cys residue in the target protein. In contrast, N-homocysteinylation involves the covalent and irreversible attachment of Hcy to a Lys residue, a reaction facilitated by HTL [61]. Notably, the S-homocysteinylation bond is relatively unstable, whereas N-homocysteinylation results in a more stable and irreversible modification, leading to permanent alterations in protein structure and function (Figure 3) [62,63,64,65].

S-homocysteinylation is a reversible modification that involves Cys residues, leading to alterations in the native structure of proteins. However, its precise implications for health and disease remain largely unclear [53,61]. The extent of S-homocysteinylation appears to be limited to a small subset of proteins, primarily due to early analytical challenges in detecting this modification. Subsequent studies have shown that Hcy can attach to proteins via disulfide bonds, a process that becomes detectable following treatment with 2-mercaptoethanol [65]. Moreover, exogenous Hcy has been found to bind to proteins and can be quantitatively measured after 2-mercaptoethanol administration. These findings provided the first direct evidence of protein S-homocysteinylation [65,66].

N-homocysteinylation is an irreversible, non-enzymatic acylation process that involves the covalent attachment of Hcy to Lys residues of proteins, such as albumin, collagen, and fibrinogen [67,68]. This modification plays a significant role in contributing to the total Hcy levels in plasma [69,70].

The formation of N-homocysteinylated proteins (N-Hcy-proteins) occurs through two main steps. First, Hcy is enzymatically converted into HTL by methionyl-tRNA synthetase (MetRS) (Figure 3) [71]. Second, HTL reacts with Lys residues on proteins, leading to the formation of N-Hcy-proteins. Additionally, two less common mechanisms of Hcy modification have been identified in humans: (1) the generation of S-nitroso-Hcy via a NO-mediated reaction [72], and (2) the demethylation of Met residues in proteins to produce Hcy, a process catalyzed by copper (Cu) and iron (Fe) (Figure 3) [73]. Similar to other PTMs, homocysteinylation disrupts protein structure and function. This modification can promote protein cross-linking, aggregation, and the generation of autoantibodies against homocysteinylated proteins, triggering immune activation [74]. Consequently, homocysteinylation is increasingly recognized as a key contributor to protein damage and its broader implications in disease pathogenesis.

Structurally and functionally, homocysteinylation alters protein properties [75]. The incorporation of Hcy modifies the chemical environment of the binding site, leading to changes in the protein’s three-dimensional conformation. In fact, Hcy binding can disrupt the spatial organization of the protein, resulting in loss of function or increased susceptibility to degradation [76]. Moreover, the presence of Hcy may alter protein reactivity by blocking or reducing its ability to interact with other molecules or execute its biological role [77].

## 4. Fibrinogen

Fibrinogen is a 340 kDa glycoprotein that plays a crucial role in the haemostatic process and is normally present in human plasma at a concentration of approximately 1.5–4 g/L. Based on its X-ray diffraction model, first described by Bailey in 1943, fibrinogen has been classified as a fibrous protein with a molecular length of 45 nm [78,79].

The liver is the primary site of plasma fibrinogen synthesis, where it is produced by hepatocytes and subsequently secreted into the bloodstream, circulating with a half-life of approximately three days. While the majority of human fibrinogen is found in plasma, significant amounts are also present in platelets, lymph, and interstitial fluid. Additionally, fibrinogen synthesis has been observed in certain extrahepatic tissues, including the bone marrow, brain, and lungs [78,80].

Fibrinogen is a hexameric glycoprotein composed of six polypeptide chains: two Aα, two Bβ, and two γ chains, consisting of 610, 461, and 411 amino acid residues, respectively [81]. These chains are held together by 29 disulfide bonds, forming a symmetrical molecule with three distinct structural regions: the central E region and two lateral D regions [78]. The D domains are each linked to the E domain via two α-helical coiled-coil segments [79]. The E domains contain the N-terminal regions of the polypeptide chains, while the D domains house the Bβ- and γ-chain regions, which form the β-nodule and γ-nodule, respectively [82]. The carboxy-terminal regions of the Aα chains form the αC domains, which exhibit greater flexibility and mobility compared to other regions [78,79,83,84].

Each fibrinogen molecule also contains four oligosaccharide chains linked via N-glycosidic bonds. These carbohydrate modifications significantly enhance fibrinogen’s solubility and influence clot structure. Fibrin formation occurs through the enzymatic cleavage of the N-terminal ends of the Aα and Bβ chains, releasing fibrinopeptide A (FpA) and fibrinopeptide B (FpB), which are located within the E domain [85]. The conversion of fibrinogen into monomeric fibrin can be represented by the reaction: (Aα Bβ γ)_2_ → (α β γ)_2_ + 2FpA + 2FpB [86].

Fibrinogen is an acute-phase protein that is upregulated in response to injury and inflammation, with its blood concentration increasing up to tenfold under such conditions [80]. This upregulation is primarily mediated by IL-6 and other pro-inflammatory cytokines, which activate intracellular signaling pathways in hepatocytes and regulate gene expression through various transcription factors [78].

Beyond its central role in hemostasis, fibrinogen is also essential for wound healing, inflammation, angiogenesis, and several other biological processes [87].

In hemostasis, fibrinogen is crucial during the initial phase, known as ‘primary hemostasis’, which involves platelet aggregation and the formation of a platelet plug at the site of vascular injury. Platelets bind to specific residues within the carboxyl-terminal regions of the fibrinogen γ chains via their integrin receptor, αIIbβ3, facilitating platelet–platelet interactions and aggregation [83].

Fibrinogen plays a crucial role in secondary hemostasis, where it is converted from a soluble macromolecule into an insoluble fibrin clot by thrombin, a serine protease. Thrombin is activated through a cascade of enzymatic reactions triggered by vessel wall injury, activated blood cells, or contact with a foreign surface [78,88,89]. The formation of a stable fibrin clot is essential to preventing blood loss and to facilitating wound healing. The polymerization of fibrinogen into fibrin is a complex, multi-step process in which each stage can significantly influence the structural and functional properties of the clot [90,91]. This process unfolds in two main stages: an enzymatic phase followed by a non-enzymatic phase. In the enzymatic stage, thrombin cleaves FpA and FpB from the Aα and Bβ chains of fibrinogen, resulting in the formation of fibrin monomers. These monomers then spontaneously assemble during the non-enzymatic phase to form protofibrils. The protofibrils elongate and laterally aggregate into fibrin fibers, which branch out to create a three-dimensional fibrin network. Finally, the clot is stabilized through covalent crosslinking, a reaction catalyzed by Factor XIIIa, a thrombin-activated transglutaminase [78].

Following clot formation, fibrin clots are degraded by the fibrinolytic system, a series of enzymatic reactions that regulate clot dissolution [78]. The primary enzyme responsible for fibrinolysis is plasmin, a serine protease derived from its inactive precursor, plasminogen, a circulating plasma zymogen. Plasminogen activation is mediated by two key serine proteases: urokinase plasminogen activator (u-PA) and tissue plasminogen activator (t-PA) [92]. Fibrinolysis primarily occurs through the conversion of plasminogen to plasmin on the fibrin surface, followed by the degradation of fibrin by plasmin, ensuring proper clot resolution and vascular integrity. In vivo, a delicate balance exists between coagulation, the conversion of fibrinogen to fibrin, and fibrinolysis. Any disruption of this equilibrium can lead to severe consequences: excessive fibrinolysis may result in bleeding, whereas the hyperactivation of coagulation can lead to thrombosis, the formation of a thrombus that obstructs blood flow within a vessel. Thrombosis is a major cause of MI, ischemic stroke, deep vein thrombosis, and other CVDs [93].

### Fibrinogen and PTMs

Fibrinogen is a complex protein that exists in various forms among healthy individuals due to genetic polymorphisms, alternative mRNA splicing, environmental factors, and PTMs [94]. PTMs—including acetylation, carbamylation, citrullination, glycation, glycosylation, homocysteinylation, methylation, nitration, oxidation, phosphorylation, and sulphation—can alter the biochemical properties of fibrinogen, significantly impacting its functional role in coagulation and fibrinolysis [95,96,97].

Oxidation and nitration, hallmark modifications linked to oxidative stress, significantly influence fibrin polymerization dynamics, promoting the formation of compact, mechanically robust fibrin networks. Glycosylation and non-enzymatic glycation induce structural alterations in fibrinogen that modulate fibrin clot architecture, frequently enhancing clot density and reducing susceptibility to fibrinolysis, particularly in hyperglycemic states such as diabetes mellitus. Acetylation and phosphorylation, including those modulated pharmacologically (e.g., via acetylsalicylic acid), impact clot ultrastructure by regulating fibrin fiber diameter and network porosity. While citrullination and homocysteinylation remain less extensively characterized, emerging evidence implicates these modifications in the dysregulation of fibrin formation and stability in the context of autoimmune and cardiovascular pathologies, respectively [96,97,98,99,100,101,102,103,104].

PTMs are pivotal in the pathophysiology of thrombotic disorders as they modulate coagulation kinetics, fibrin clot architecture, fibrinolytic susceptibility, and can directly contribute to thrombus formation and disease progression [96,105,106,107,108,109,110]. PTM-induced structural changes in fibrin clots critically influence their viscoelastic properties, which in turn affect hemostatic balance [97,111]. Notably, elevated clot stiffness has been robustly correlated with an increased incidence of thrombotic events [112]. Fibrin networks composed of thicker fibers, reduced branching, and enlarged pores exhibit greater permeability and are more amenable to fibrinolysis. In contrast, clots characterized by thinner fibers, enhanced branching, and smaller pore sizes demonstrate diminished permeability and heightened resistance to plasmin-mediated degradation, thereby fostering a prothrombotic milieu [95,113]. Advancing our mechanistic understanding of fibrinogen PTMs holds significant promise for the refinement of therapeutic strategies targeting thrombotic diseases.

## 5. Fibrinogen Homocysteinylation and Clinical Consequences

The N-homocysteinylation of fibrinogen is a non-enzymatic post-translational modification mediated by HTL, a reactive cyclic thioester formed through the misactivation of homocysteine by methionyl-tRNA synthetase [114]. HTL selectively reacts with the ε-amino groups of lysine residues, forming covalent amide linkages and introducing a bulky homocysteine side chain bearing a free thiol group. This modification disrupts local electrostatic interactions and protein conformation. In fibrinogen, twelve lysine residues have been identified as sites of N-homocysteinylation, including γLys380, γLys381, and γLys385 in the γ-chain D-domain; BβLys58 and BβLys344 in the Bβ chain; and seven residues in the Aα chain, notably AαLys448, AαLys508, AαLys539, AαLys556, AαLys562, AαLys572, and AαLys583—all of which are clustered in the αC region [114]. These regions are known to play critical roles in fibrin monomer polymerization, protofibril lateral association, and cross-linking mediated by activated Factor XIII (FXIIIa) [115]. The homocysteinylation-induced structural perturbations result in fibrin clots with thinner, more densely packed fibers and significantly increased resistance to plasmin-mediated fibrinolysis, even though the binding affinity for tissue plasminogen activator (tPA) and plasminogen remains intact or slightly enhanced. However, the efficiency of plasminogen activation is reduced, likely due to the conformational misalignment of cleavage sites [114]. Moreover, platelet adhesion to HTL-modified fibrinogen is impaired, particularly via disruption of the RGD (arginine-glycine-aspartic acid) motif in the Aα chain—a key integrin αIIbβ3 recognition site. Homocysteinylation near or within this motif may sterically hinder its exposure or alter its conformation, compromising fibrinogen’s ability to support platelet aggregation [116,117]. These effects, compounded by altered calcium-binding dynamics, mirror features of inherited dysfibrinogenemias and support a mechanistic link between hyperhomocysteinemia and heightened thrombotic risk [114,115,118,119,120,121,122,123,124,125].

In human fibrinogen, three specific Lys residues—AαLys562, BβLys344, and γLys385—have been identified as primary targets of homocysteinylation in both in vitro and in vivo studies [126]. This modification impairs fibrinogen functionality, reinforcing the link between HHcy and an increased risk of thrombosis and atherosclerosis [115,127,128,129,130].

A recent study [131] evaluated plasma and urinary levels of sulfur-containing amino acid metabolites and fibrin clot properties in stroke patients and healthy individuals. In an earlier large-cohort study of coronary artery disease patients [132], researchers found that urinary HTL (uHTL) and plasma Cys (pCys) were associated with fibrin clot properties and served as predictors of MI. In the study by Sikora et al., certain metabolites (e.g., uHTL, urinary glutathione [uGSH], and plasma cysteinylglycine [pCysGly]) were found to directly influence clot properties. In contrast, other metabolites (e.g., urinary homocysteine [uHcy], urinary Cys [uCys], and pCys), along with genetic factors such as the MTHFR C677T polymorphism, were associated with stroke risk independently of clot alterations. Both studies reached the same conclusion: targeting sulfur-containing amino acid metabolites and their excretion may provide a therapeutic strategy to mitigate prothrombotic risks and reduce stroke incidence.

The studies reviewed here examine the effects of HHcy, characterized by elevated plasma Hcy levels, on fibrin polymerization and fibrinolysis (Table 1). Among these, only seven studies were specifically considered for clot structure analysis (Table 2).

As summarized in Table 1, in vitro investigations into fibrinogen homocysteinylation—typically through incubation with varying concentrations of Hcy or HTL—have yielded heterogeneous results regarding clotting kinetics. Sauls et al. reported that hyperhomocysteinemic conditions led to decreased fibrin polymerization rates, prolonged lag phases, and reduced maximal turbidity, indicative of impaired fibrin assembly. Notably, reptilase time was prolonged in plasma from Hcy-treated rabbits, while thrombin clotting time was shortened, suggesting altered fibrinogen interactions with coagulation enzymes [115]. However, subsequent studies by the same group did not further assess polymerization kinetics, leaving uncertainties regarding reproducibility [114,122]. Conversely, Lauricella et al. observed no major changes in polymerization rates upon Hcy exposure, although increased maximum absorbance suggested the formation of denser fibrin networks [118,119]. Marchi et al. noted significant effects only at higher Hcy concentrations (>50 µM), including slower polymerization, a delayed lag phase, and reduced turbidity [121]. Genoud et al. similarly found diminished clot formation and reduced final turbidity in HTL-treated samples, yet SEM analysis indicated increased clot compactness [124]. In contrast, Malinoska et al. reported enhanced fibrin polymerization, with elevated turbidity in plasma exposed to oxidized or reduced Hcy, though structural characterization was lacking [123].

These discrepancies likely stem from methodological variability, including the form of Hcy (reduced, oxidized, or HTL), the biological matrix (purified fibrinogen vs. plasma), incubation conditions, and the often supraphysiological Hcy concentrations used. While purified systems allow the direct assessment of biochemical effects on fibrinogen, plasma introduces complexity via interactions with coagulation proteins and inhibitors such as albumin. The use of high Hcy concentrations (>100 µM) may limit physiological relevance.

Despite inconsistencies in clot formation parameters, a recurring observation across nearly all studies is a marked reduction in fibrinolytic efficiency following homocysteinylation [114,115,119,120,121,122,123,125]. This is frequently associated with the development of abnormally dense fibrin clots, characterized by compact architecture and altered fiber morphology, rendering them more resistant to plasmin-mediated degradation. Impaired fibrinolysis contributes to a shift in hemostatic balance favoring thrombosis, with clear clinical implications.

The literature on fibrinogen homocysteinylation remains limited and methodologically diverse. Only a subset of studies has examined fibrin clot structure directly using SEM—such as those by Lauricella et al. [118,119], Sauls et al. [114,115], and Genoud et al. [124]—consistently reporting the formation of denser networks composed of shorter and more tightly packed fibers. However, these findings were derived from in vitro models often employing supraphysiological Hcy concentrations. Other studies, including that by Undas et al. [120], inferred structural alterations indirectly by assessing clot permeability and lysis times in clinical samples. Experimental models ranged widely—from purified fibrinogen and platelet-poor plasma to animal models and human subjects—further contributing to heterogeneity in outcomes (Table 2).

Nevertheless, increased clot density and decreased permeability emerge as consistent features, indicative of a prothrombotic phenotype. These properties not only impede fibrinolytic enzyme penetration but also enhance resistance to clot breakdown, potentially increasing the risk of thromboembolic events. Given the paucity of mechanistic studies, particularly those evaluating secondary and tertiary structural effects of homocysteinylation, further research is warranted.

Importantly, oxidative stress appears to intersect with Hcy-mediated effects. Oxidative modifications of fibrinogen can disrupt residues critical for polymerization, enzyme binding, and clot stability. Our group has previously shown that oxidative stress induces both structural and functional alterations in fibrinogen, resulting in impaired fibrinolysis and a prothrombotic state. In liver transplant recipients, oxidative fibrinogen modifications were linked to elevated cardiovascular risk, underscoring the clinical relevance of redox-mediated dysregulation [109]. Similarly, studies in the subacute phase of MI have demonstrated associations between oxidatively modified fibrinogen and altered clot architecture and function [110].

These findings suggest that Hcy-induced oxidative stress—either directly or through reactive intermediates like HTL—may represent a mechanistic bridge between metabolic disturbances and thrombotic risk. This aligns Hcy with other cardiovascular risk factors (e.g., smoking, diabetes, chronic inflammation) that similarly promote oxidative alterations in fibrinogen. Elucidating these converging pathways could inform the development of targeted interventions aimed at mitigating redox-driven fibrin dysfunction and reducing thrombotic complications in at-risk populations.

Recent studies have also highlighted the broader clinical implications of fibrinogen homocysteinylation beyond thrombosis [133,134]. For example, plasma and urinary levels of sulfur-containing amino acid metabolites, such as homocysteine, cysteine, and cysteinylglycine, have been proposed as emerging biomarkers not only for cardiovascular risk but also for neurological conditions, including cognitive decline and Parkinson’s disease [135,136]. The MTHFR C677T polymorphism, which compromises the remethylation of homocysteine to methionine, has been robustly associated with increased plasma homocysteine levels and thrombotic risk, as well as poorer neurological outcomes [137,138,139,140]. These findings support the potential use of homocysteinylation-related markers for early risk stratification in both vascular and neurodegenerative disorders.

## 6. Conclusions and Future Prospects

Homocysteinylation constitutes a critical post-translational modification of fibrinogen, with significant implications for coagulation dynamics and thrombotic pathophysiology. The reviewed evidence underscores that elevated Hcy levels can perturb fibrinogen structure and function, resulting in altered clot architecture, enhanced mechanical stability, and reduced susceptibility to fibrinolysis—hallmarks of a prothrombotic phenotype. Despite extensive investigation, discrepancies remain, particularly regarding fibrin fiber diameter and polymerization kinetics, highlighting the need for further elucidation of the molecular mechanisms underlying these effects. Moreover, the consequences of homocysteinylation on the secondary and tertiary structure of fibrinogen remain largely undefined.

To enhance the reproducibility and interpretability of findings, future studies should implement standardized experimental frameworks, including uniform concentrations of Hcy or HTL, consistent use of either plasma or purified fibrinogen systems, and harmonized clotting and lysis assays. Such methodological consistency would facilitate cross-study comparisons and improve the robustness of observed outcomes.

Advancements in analytical technologies, particularly mass spectrometry-based proteomics, offer a promising approach to precisely identify homocysteinylated Lys residues on fibrinogen and assess the conformational consequences of these modifications. In parallel, high-resolution imaging modalities such as cryo-electron microscopy and atomic force microscopy could provide detailed structural insights into fibrin network alterations induced by homocysteinylation.

Integrating mechanistic data with clinical observations will be essential to delineating the role of fibrinogen homocysteinylation across diverse thrombotic and cardiovascular conditions. Such efforts may reveal differential effects in specific patient cohorts and support the identification of homocysteinylation-based biomarkers for diagnostic or prognostic applications. A deeper understanding of the long-term hemostatic consequences of HHcy could inform early risk stratification and intervention strategies.

Therapeutically, targeting homocysteinylation through specific inhibitors or modulators may offer a novel strategy to mitigate thrombotic risk in patients with elevated Hcy levels, potentially preserving physiological hemostasis while preventing pathological clot formation.

Beyond thrombosis, homocysteinylation has emerged as a biomarker in neurological conditions, including Parkinson’s disease and cognitive impairment, due to its contribution to protein dysfunction and neurotoxicity. This broader relevance suggests its potential utility as a biomarker in a range of pathological states. Given its demonstrated impact on fibrinogen structure and function, homocysteinylation may also serve as a valuable marker for early detection and personalized management of thrombotic disease.

Finally, the interplay between homocysteinylation and other PTMs—such as glycosylation and phosphorylation—remains an underexplored yet promising avenue. Investigating whether these modifications exhibit synergistic or antagonistic effects on fibrinogen functionality could advance our understanding of complex coagulation networks. When combined with systems biology approaches and personalized medicine strategies, such insights may pave the way for targeted, patient-specific therapeutic interventions that improve clinical outcomes in HHcy-associated disorders.

A major challenge in the field remains the methodological heterogeneity across studies. Inconsistencies in clot structure and polymerization kinetics are largely attributable to variations in experimental design, including the form and concentration of homocysteine or HTL used, the biological matrix (e.g., purified fibrinogen vs. platelet-poor plasma), and analytical methods (e.g., turbidity assays vs. SEM). To address this, we recommend the adoption of standardized protocols encompassing (i) physiologically relevant concentrations of homocysteine (10–50 µM); (ii) consistent use of either purified fibrinogen or well-characterized plasma matrices; and (iii) uniform clotting and fibrinolysis assays to enable reliable cross-study comparisons.

Additionally, the translational potential of homocysteinylated fibrinogen as a disease biomarker or therapeutic target warrants further investigation. Mass spectrometry-based profiling and imaging approaches could facilitate the identification of specific modification sites and their clinical relevance, paving the way for novel diagnostics and precision therapies.

## Figures and Tables

**Table 1:** Articles about fibrinogen functional analysis.

	Fibrinogen Analysis			
Author	Model	Method	Polymerization	Fibrinolysis
Lauricella et al. (2002) [118]	In vitro	Plasma + 300 μM Hcys, Cys, Hcyst	=	Probably -
Sauls et al. (2003) [115]	In vivo (rabbit model)	Plasma or fibrinogen	− with reptilase + with thrombin	_
Lauricella et al. (2006) [119]	In vitro	Plasma + 500 μM Hcys	= + Max Abs	_
Sauls et al. (2006) [114]	In vitro	Fibrinogen + 300 μM HTL	nd	_
Undas et al. (2006) [120]	In vitro (human)	Plasma + Hcy	nd	_
Marchi et al. (2008) [121]	In vitro	Plasma + 13, 19, 52 μM Hcy	− + Lag phase − Max Abs	_
Marchi et al. (2008) [121]	In vitro	Plasma + 251 μM Hcy	= = Lag phase = Max Abs	nd
Marchi et al. (2008) [121]	In vitro	Fibrinogen + 408 μM Hcy	− + Lag phase − Max Abs	nd
Sauls et al. (2011) [122]	In vitro	Fibrinogen + 300 μM HTL	nd	_
Malinowska et al. (2011) [123]	In vitro	Plasma + 0.1–1 mM HTL	+	_
Genoud et al. (2014) [124]	In vitro	Fibrinogen + 100, 500 and 1000 μmol/L HTL	− + Lag phase − Max Abs	nd
Cellai et al. (2014) [125]	Clinical trial—patients with a history of PE	Plasma	nd	_

The table provides an overview of the experimental models, methodologies, and key findings from the analyzed studies, specifically regarding fibrin polymerization and fibrinolysis outcomes. Symbols used include “=”, indicating no observed change; “+”, denoting an increase in the respective parameter; “−”, indicating a decrease; and “nd”, representing parameters not assessed within the study. Abbreviations: PE, pulmonary embolism; HTL, homocysteine thiolactone.

**Table 2:** Articles about clot structural analysis.

Clot Analysis		
Author	Method	Clot Analysis
Lauricella et al. (2002) [118]	Analysis of fibrin networks by electronic microscopy	+ fiber diameter + density + branched + shorter
Sauls et al. (2003) [115]	SEM of plasma clots	− fiber diameter + shorter + density
Lauricella et al. (2006) [119]	Electron microscopy	+ fiber diameter + density
Sauls et al. (2006) [114]	Mass spectrometric analysis	− fiber diameter + density
Undas et al. (2006) [120]	Fibrin permeation analysis	+ density
Marchi et al. (2008) [121]	SEM	= fiber diameter
Genoud et al. (2014) [124]	SEM	− fiber diameter + density

The table summarizes the methodologies employed in the analyzed studies for clot characterization. Symbols are defined as follows: “=” indicates no change in the assessed parameter; “+” denotes an increase; “−” signifies a decrease; and “nd” indicates the parameter was not determined or evaluated. Abbreviation: SEM, scanning electron microscopy.

## Abbreviations

CVDs	Cardiovascular diseases
Cys	Cysteine
Hcy	Homocysteine
HHcy	Hyperhomocysteinemia
HTL	Homocysteine-thiolactone
ICAM-1	Intracellular adhesion molecule-1
IL-1β	Interleukin-1β
IL-6	Interleukin-6
Lys	Lysine
Met	Methionine
MI	Myocardial infarction
N-Hcy-proteins	N-homocysteinylated proteins
NMDA	N-methyl-D-aspartate
NO	Nitric Oxide
PTMs	Post-translational modifications
ROS	Reactive Oxygen Species
SAM	S-adenosylmethionine
SAH	S-adenosylhomocysteine
TLR4	Toll-like receptor 4
TNF-α	Tumor necrosis factor-alpha
